# Psychosocial factors and distress: a comparison between ethnic Norwegians and ethnic Pakistanis in Oslo, Norway

**DOI:** 10.1186/1471-2458-6-182

**Published:** 2006-07-10

**Authors:** Hammad Raza Syed, Odd Steffen Dalgard, Ingvild Dalen, Bjørgulf Claussen, Akthar Hussain, Randi Selmer, Nora Ahlberg

**Affiliations:** 1 Department of International Health, Institute of General Practice and Community Medicine, University of Oslo, Norway; 2 National Centre for Minority Health Research, (NAKMI), Ullevaal University Hospital, Oslo, Norway; 3 Department of Mental Health, Norwegian Institute of Public Health, Oslo, Norway; 4 Department of Biostatistics, Institute of Basic Medical Sciences, University of Oslo, Norway; 5 Department of Epidemiology, Norwegian Institute of Public Health, Oslo, Norway

## Abstract

**Background:**

In the Norwegian context, higher mental distress has been reported for the non-Western immigrants compared to the ethnic Norwegians and Western immigrants. This high level of distress is often related to different socio-economic conditions in this group. No efforts have been made earlier to observe the impact of changed psychosocial conditions on the state of mental distress of these immigrant communities due to the migration process. Therefore, the objective of the study was to investigate the association between psychological distress and psychosocial factors among Pakistani immigrants and ethnic Norwegians in Oslo, and to investigate to what extent differences in mental health could be explained by psychosocial and socioeconomic conditions.

**Method:**

Data was collected from questionnaires as a part of the Oslo Health Study 2000–2001. 13581 Norwegian born (attendance rate 46%) and 339 ethnic Pakistanis (attendance rate 38%) in the selected age groups participated. A 10-item version of Hopkins Symptom Checklist (HSCL) was used as a measure of psychological distress.

**Results:**

Pakistanis reported less education and lower employment rate than Norwegians (p < 0.005). The Pakistani immigrants also reported higher distress, mean HSCL score 1.53(1.48–1.59), compared to the ethnic Norwegians, HSCL score 1.30(1.29–1.30). The groups differed significantly (p < 0.005) with respect to social support and feeling of powerlessness, the Pakistanis reporting less support and more powerlessness. The expected difference in mean distress was reduced from 0.23 (0.19–0.29) to 0.07 (0.01–0.12) and 0.12 (0.07–0.18) when adjusted for socioeconomic and social support variables respectively. Adjusting for all these variables simultaneously, the difference in the distress level between the two groups was eliminated

**Conclusion:**

Poor social support and economic conditions are important mediators of mental health among immigrants. The public health recommendations/interventions should deal with both the economic conditions and social support system of immigrant communities simultaneously.

## Background

### Migration and mental health

Migration that deals with the moving of people from one particular geographical area to another has long been under investigation in relation to its impact on mental health of the migrating people [1-4]. It has been argued that consequences of migration and resettlement pose certain threats to the psychological well-being of the immigrants due to accompanied changes in their physical and psychosocial environment [5-7]. The psychosocial factors that might be influenced by migration, and thereby pose a negative effect on mental health, are social support, social participation and feeling of powerlessness.

In general, evidence suggests that social support deals with the "resources provided by other persons" and involves both interactions and transactions between people. Social support provides a direct and indirect buffering effect, which moderates the impact of stress on health [8]. Social participation is another psychosocial factor that has been extensively studied in relation to health and survival [9]. Good social support and high level of social participation influence mental health through giving meaning and coping resources to one's life and subsequently reducing distress [10]. Powerlessness, a less studied phenomenon in relation to mental health, has two dimensions: the psychosocial dimension deals with self-esteem, self-trust and self-efficacy, whereas the social action oriented dimension involves control over one's own life situations [11]. Our previous knowledge based on empirical studies related to empowerment has shown that it is positively associated with perceived quality of life [12], self-esteem and social support [13] and negatively associated with the severity of psychiatric symptoms [14].

In line with the assumptions above, it has been observed that immigrants who were subjected to changed psychosocial environment in terms of low social support, changed patterns of social participation or lack of control over their life events in a new society, exhibit higher level of psychological symptoms [15,16]. Hence, it can be assumed that migration by itself does not constitute a threat to the mental health of immigrants, but changes in psychosocial factors might be the important mediators in the pathway between migration and mental health status [4,17-19]. This might be the reason that studies dealing with acculturation have reported higher distress and depressive symptoms for those immigrant communities who migrate to culturally and socially distinct societies and try to adapt to the new social circumstances after migration [17,20,21].

A higher distress level has been reported among non-Western immigrants in Oslo [22] and adolescents with non-Western immigrant background [23]. Based on the same sample as used in the present study, a recent paper (in press), reported higher levels of psychological distress among immigrants from low income countries compared to Norwegian born, with employment, social support and powerlessness as important explanatory factors [24].

### Pakistanis in Norway

The Norwegian migration history is agreed upon the fact that men with Pakistani background were among the earliest guest workers who came to Norway in the late 1960's [25]. This was the time when after the discovery of oil, Norwegian economy was booming and there was a great need for unskilled labor in Norway. Another important characteristic of this migration was that most of the Pakistanis, who migrated to Norway, belonged to one district in Pakistan, i.e. Gujarat. Moreover, it was mostly male dominated migration from that district due to pull and push factors. After the enforcement of immigration legislation in 1975 in Norway, trend in migration changed and family reunification from this district became the main motivating factor for the migration to Norway.

Although, Pakistani immigrants now constitute more than 8% of the total immigrant population in Norway, no previous study, however, has focused specifically on mental health and psychosocial factors among this largest non-Western immigrant group in Norway, and our knowledge regarding the psychosocial conditions of different ethnic communities is sparse. It is not known to what extent variables like social support, social participation and feeling of powerlessness relate to mental health in the same way in the Pakistanis and Norwegians born in Oslo and it is not known to what extent the differences in mental health between the two ethnic groups can be explained by these psychosocial conditions. So this will be the objective of this study.

## Methods

### Sample

A general health survey known as the Oslo Health Study was jointly organized during 2000–2001 by the University of Oslo, National Health Screening Services of Norway (now the Norwegian Institute of Public Health), and Oslo Municipality. The study subjects were all the inhabitants of Oslo born in 1970, 1960, 1955, 1940/41 and 1924/25. Of the total 40, 888 in the sample, only 18,770 (45.9%) participated. The response rate for ethnic Norwegian was 46% and for Pakistanis it was 38%(of the total 884 persons recruited for the study, 339 actually participated). However, the weighted prevalence of self-rated health and other analyzed variables differed only slightly between attendees and the target population, and the association measures were found to be less influenced by the self selection. The details on the methodology were described elsewhere [26]. The Norwegian Registry of Vital Statistics provided information on age, gender, country of birth and residential address. Ethnicity was determined on the basis of country of birth from this register. A cross check with Statistics Norway (SSB) registers confirmed that in 99.8% of the cases the country of birth was identical to their 'country of origin' [27].

The study protocol was reviewed and approved by the Regional Committee for Medical Research Ethics in Norway. The ethical aspects were in full agreement with the Helsinki declaration.

### Research design

The letter of invitation included a questionnaire to be filled in at home and brought to the screening site. Questions about socioeconomic conditions (education, employment status and household income) and psychosocial factors (social support, social participation, powerlessness and psychological distress) were included. Except the factors included in powerlessness, all other variables were translated into Urdu – the native language of the Pakistanis. Moreover, invitation letter and information about the study was also provided in 'Urdu' to the ethnic Pakistani participant. In addition, written consent was secured from the participants in their native languages.

### Instruments

#### Psychological distress (HSCL-10)

A 10-item version of Hopkins Symptom Checklist was used as a measure of psychological distress [28]. Each item was rated on a scale of 1 (not at all) to 4 (extremely) during the past week. In contrast to the 25 items Hopkins Symptoms Checklist (HSCL-25), where symptoms can be subdivided into depression and anxiety categories, the HSCL-10 provides a general measure of psychological distress [28]. High internal consistency of this instrument is reported (Cronbach's alpha = 0.89), with no difference with respect to ethnic Norwegians and immigrants [24]. The internal consistency of this scale was even high when estimated for ethnic Pakistanis in our study, i.e. Cronbach's alpha 0.91. Only those respondents who answered at least 6 of the 10 items were included in the study and data for unanswered items were imputed using the mean value of the items reported by the respondents and we used the mean score of this scale in our analysis. By this criterion 591 ethnic Norwegians and 90 Pakistanis were not included in this study as they had more than four missing values for HSCL-10.

#### Social support

Social support was measured by two questions in the Oslo social support scale [29]:

*a. How many good friends do you have? *(*Count those whom you can take to in confidence and who can help you when you need help. Do not count those who you live together with, but include other relatives*.)

b. How much interest do people show in the things you do?

The response to question a. was recorded as total number of friends and this response was categorized into 0–10 categories. Whereas response to question b. was recorded against the following values: Great interest = 4, some interest = 3, slight interest = 2, no interest/uncertain = 1.

#### Social participation

The following questions were asked to register the level of social participation among the respondents:

a. How many societies, clubs, groups, congregations and etc. do you take part in your free time?

b. Do you feel that you can influence what happen in the local community where you live?

The response to the first question was recorded as the total number reported by the respondents and 0–10 categories were created from this response. The response to question b. had the four values: yes, to a larger degree = 4, yes, to some degree = 3, yes, to slight degree = 2 and no = 1.

#### Powerlessness

Out of the 28 items of the Empowerment scale developed by Rogers et al. [13], the variables related to the concept of Power-powerlessness was used in this study. The following questions were used;

a. I feel powerless most of the time

b. Making waves never gets you anywhere

c. You can't fight city hall

d. When I am unsure about something, I usually go along with the group

e. Experts are in the best position to decide what people should do and learn

f. Most of the misfortunes in my life were due to bad luck

g. Usually I feel alone

h. *People have no right to get angry just because they don't like something*

The response to each variable had the following values: strongly disagree 1, somewhat disagree 2, somewhat agree 3, and strongly agree 4. The item scores were summarized as an index. Those respondents who answered at least 4 out of 8 questions were included in the analysis, and data for unanswered items were imputed using the mean value of the items reported by the respondent.

The internal consistency for this scale was almost equal for the ethnic Norwegians and Pakistanis in the current study, Cronbach's alpha being 0.60 and 0.61 respectively. For Norwegians its value remained consistent even after step wise exclusion of its items, where as a modest improvement was seen after the exclusion of items, i.e. "making waves never gets you anywhere" and "people have no right to get angry just because they don't like something" for ethnic Pakistanis. The corresponding Cronbach's alpha was 0.63 and 0.64.

### Statistical analyses

Pearson's χ^2 ^test, analysis of variance (ANOVA) and linear regression analysis were used. All the p values reported are from two-tailed tests and level of significance was set at 0.05. Data were analyzed by using Statistical Package for Social Sciences (SPSS) – version 13.

## Results

All the indices of socio-demographic and SE characteristics differed significantly (p < 0.001) between the participants from these two groups. The ethnic Pakistanis were younger, more often married, had lower education, were less often in paid jobs (Table 1) compared to Norwegians.

Pakistani respondents also reported lower number of friends, less interest from others in their activities, less participation in the social gatherings and higher level of powerlessness compared to the Norwegians. These differences were statistically significant both unadjusted and when adjusted for age and sex. In contrast, ethnic Pakistanis reported significantly higher influence on the local community compared to Norwegians (Table 2).

While looking into the association between SE factors and the psychosocial variables in these two ethnic communities, significant interactions emerged. Interaction analysis indicated differences between Pakistanis and Norwegians for the effects of education (p < 0.000) and employment status (p = 0.001) on distress. In Norwegians, psychological distress improved with the increase in education level but no such association was observed for ethnic Pakistanis. Where as employment status was related to reduce psychological distress in ethnic Norwegians, but being employed was not related to any significant reduction in distress among ethnic Pakistanis.

The overall mean distress (adjusted for age and sex) for ethnic Pakistanis was 1.53 (95% CI: 1.48–1.59) and 1.30 (95% CI: 1.29–1.30) for ethnic Norwegians. All the psychosocial variables showed significant association with psychological distress for ethnic Norwegians. In the case of ethnic Pakistanis, significant associations were observed with number of good friends and powerlessness (Table 3). Interaction analyses further indicated that association with number of good friends was significantly stronger (p = 0.002) for ethnic Pakistanis than in Norwegians. With respect to others' interest and influence on the local community, these associations were on the same level in the two groups. Opposite to the findings in the Norwegians, participation in groups for ethnic Pakistanis was associated with increased level of psychological distress (p < 0.000).

Adjusting for age and sex, the expected difference in distress between ethnic Pakistanis and Norwegians was 0.23 (0.19–0.29), with Pakistanis having most distress. This difference was reduced to 0.07 (0.01 to 0.12) and 0.12 (0.07 to 0.18) when adjusted for SE indicators (education and employment status) and for social support (number of good friends and people's interest in your activity), respectively. The differences, however, were still statistically significant. Adjusting for all of these variables simultaneously eliminated the difference in distress between ethnic Pakistanis and Norwegians (Table 4).

## Discussion

The differences observed between these two ethnic communities with regard to the socio-demographic characteristics were in accordance with the already existing statistics in Norway. For example, we know that immigrant population in general is younger than the total population of Norway. 50% of the immigrant population is in the age group 20–44 years compared to 35% of the total population [30]. Likewise, existing information regarding employment rate indicates that, Pakistani and Turkish immigrants have lowest employment rate (44%) compared to 69.3% in the entire population [31]. It has been further reported that the overall gap in income between non-Western immigrants and native born Norwegians is 20–30% for men and somewhat lower for women [32]. Moreover, marriage is the only socially acceptable pattern of cohabiting among ethnic Pakistanis, where as this is not the only option in case of ethnic Norwegians. The difference in educational level among ethnic Pakistanis and Norwegians in this study might be explained by the sample consisting of first generation immigrants from Pakistan, who came to Norway as young guest worker with limited or no education.

Most of the psychosocial factors in the study showed less favorable outcome for the ethnic Pakistanis than for the Norwegians. With respect to influence on the local community, however, the opposite was true. Also as expected, these factors were associated to psychological distress, but somewhat different in the two ethnic groups.

The total number of close friends was more strongly associated to psychological distress in the ethnic Pakistanis than in the ethnic Norwegians. A possible explanation for this could be that the Pakistanis in Oslo are more exposed to economic and social stressors than the Norwegian born are, and for that reason are in stronger need of friends to cope. This is in accordance with the "buffer hypothesis" of social support, and also in accordance with the earlier findings that the disruption of traditional support systems has a negative impact on the psychological well-being of the immigrants [10,33-35].

For Norwegians participation in groups was significantly and negatively associated with distress, where as for Pakistanis this association was in opposite direction. This difference in trend and association was also confirmed by the interaction analysis. One possible explanation of this difference in trend observed in this study might represent the feelings of Pakistanis while participating in social gatherings organized by the host members of the society due to some language, cultural or due to their hierarchal perception about their social position. It is hard to believe that this community feels the same when they participate in their own social gatherings. This response needs further research in future.

To feel that one has some influence on the local community was negatively associated to psychological distress in both ethnic groups, but somewhat unexpectedly, Pakistanis reported more influence than the Norwegians. This response might indicate the diversity of these two communities in terms of their social structure and meaning to this variable. It seems that while reporting their influence on the local community ethnic Pakistanis might have referred to their influence in their own community based on the personal relations and affiliations. This is in agreement with the observations reported by other studies where it has been discussed that members of Western and non-Western society's represent two different approaches of social structure, i.e. individualistic and collectivistic. In contrast to more or less equal and horizontal relations and social ties in the individualistic societies, the relations and social ties in collective societies are vertical in nature [36]. Consequently, individuals in collective societies are bound by relationships which emphasize common fate and interest. It is therefore possible that higher influence on the local community reported by Pakistanis here represent the naturally existing characteristics of their social structure based on common fate/interest or interpersonal affiliations. It might not necessarily correspond with the Western concept of community involvement/influence.

Like many other epidemiological studies, social support and health are also investigated from an egocentric approach in this study. This approach mainly deals and focuses on the structure and function of networks immediately surrounding the people ignoring the fact that social support and networks are embedded within a broader set of macro-social exchanges [37]. To account for the macro-social concept of exchanges in terms of broader social networks, we included the sense of powerlessness as a psychosocial factor in this study. Powerlessness is a feeling that arises when an individual considers him/her worthless in terms of societal norms, attitudes and human models. The literature in this field suggests that living in poverty, with low self-esteem, high demands, low level of control, chronic stress, lack of social support and lack of resources are some of the key physical and social risk factors related to the feeling of powerlessness [38,39]. Most of these risk factors are an essential part of the migration process and resettlement experience. Therefore, by reporting higher feeling of powerlessness, Pakistanis might have pointed indirectly toward difficulties in their life situation due to their immigrant status.

Moreover, results of the study show that the expected difference in the level of distress between these two communities becomes insignificant after adjusting simultaneously for psychosocial and SE factors. When all these factors are taken into consideration, it seems that there is no difference in mental health between Norwegians and Pakistanis.

As already reported by the author in another paper from the same sample [40], SE factors alone cannot explain the difference in psychological distress between the two ethnic groups. When stratifying for level of socioeconomic status, the rates of distress were still higher in the Pakistanis, and this difference was especially strong among those with high socioeconomic status, in particular among those with higher education. Whereas Norwegians with higher education displayed relatively low rates of distress, the opposite was true for the Pakistanis.

The finding that the difference in psychological distress between Norwegians and Pakistanis disappears when adjusting for SE factors as well as social support, however not when adjusting for each variable separately, indicates that both these factors are important mediators between immigration and mental health. Taken into consideration that especially Pakistanis with higher education display a relatively high level of distress, it seems that the lack of support is especially important for mental health in this group. This is in accordance with the finding that social support did not increase with increasing education among Pakistanis, contrary to the situation for Norwegians.

As a conclusion, the study shows that access to work and income are important for health, for Norwegians as well as for immigrants from Pakistan, however in addition it shows that the situation is more complex for the immigrants than for the Norwegians. It seems that immigrants with higher education, and hence with higher aspirations with respect to a social career, run into special frustrations. This may be related to a certain degree of discrimination on the labor market against the immigrants, which may be especially strong against those with higher education. There are many stories about Pakistanis with higher education in Norway, or immigrants from other culturally different countries, who apply for an endless number of white collar jobs without any positive response, as if their name in itself was a reason for discrimination. Then it becomes easier for them to get a job as a taxi-driver or housecleaner. The existing statistics from Norway confirms that 25% of the non-Western immigrants are enrolled within elementary occupations compared to 6.7% of the entire working population [31]. Moreover, it has been reported that immigrants do not perform as well on the labor market as natives with similar characteristics and a large proportion of immigrants from non-Western countries is characterized as self-employed marginalized, even when controlling for observed and unobserved individual characteristics [41].

From a methodological point of view, the weakness of the study is that it is based on a cross-sectional design. The inherent problem of a cross-sectional design is that the outcome (in this case distress) and the exposure (in this case psychosocial and socio-economic condition) are collected simultaneously and thereby preventing conclusions regarding causality. Moreover, little attention has been paid to the information bias emerging from the dependent error in the cross-sectional studies, which means a possible correlation between the degree of error in measured exposure and measured outcome. Thus, it is possible that estimated associations between distress and psychosocial factors are falsely inflated in our study [42].

The data collected by self-reporting has often raised the concerns about its validity. However, self-reported health and related psychosocial variables are widely used in European [43-45] and American studies [46,47].

Besides the validity of self-reported data, we were also concerned about the apprehension of psychological variables by the ethnic Pakistanis in Oslo. A word to word translation was made from 'Norwegian' to 'Urdu' language for all the psychosocial instruments used in this study. However the instrument for powerlessness was not even translated. It might be possible that metaphors and phrases used in those instruments are not culturally relevant to Pakistani respondents. Hence, results from these instruments must be interpreted with caution. Keeping this issue in mind, we have planned to conduct another study in Pakistan and Oslo after securing the culturally sensitive translation of instruments used in the current study. Another methodological challenge was related to the low participation rate for both the ethnic Pakistanis and Norwegians in our study. Low participation in epidemiological studies may threaten the validity and generalizability of the results due to the possibility of selective participation [48]. However, in case of the current study such an impact is negligible [26].

In addition to low participation rate, it was a problem that not all the participants answered to every question. The lowest response rate among Pakistanis was related to the total household income. It was further noticed that 86.4% of the respondents with lower household income and 80% of the respondents in the younger age group (30–45 years) had not responded to HSCL among ethnic Pakistanis, where as no such association was seen for the Norwegians.

## Conclusion

This study has revealed the importance of psychosocial factors in addition to SE factors on the psychological distress among Pakistani immigrants in Oslo. It seems that to improve the mental health of this immigrant community, we need to address both SE and psychosocial issues. This could be achieved through adopting a strategy that not only deals with better SE opportunities equivalent to their education and other socio-demographic characteristics, but also provide opportunities to bring them in contact with the main stream Norwegian society. In this way it might be possible that while gaining SE stability, this community will also find the way to reduce the burden of dispersed/reduced social support on their distress level by interacting and affiliating to the Norwegian society. It has been reported in studies that frequent interactions between immigrant communities and the individuals of the society of resettlement have a positive impact on the mental health of immigrants and vice versa [49,50].

By adopting such a strategy, it would be possible that this community would start taking an active part in the context of the broader Norwegian society. This active involvement would then reduce the prevailing feeling of powerlessness in this community. Gaining sense of empowerment in this way would enhance their abilities to meet their own needs, solve their own problems and would give them confidence to interact with the Norwegian society. In return, we can expect that prevailing prejudice in Norwegian society would reduce. This process in return will alleviate the acculturation process of this community and will give strength to the multicultural concept of Norwegian society by reducing the inequalities in health.

The second conclusion of this study is related to the importance of the more planned and solid methodological grounds to conduct research with immigrants. In comparative studies it is important that we focus on the substance of the information being compared rather than assuming that we are dealing with similar information.

Hence, we need to develop culturally sensitive and validated instruments in the field of immigrant's health in Norway.

## Competing interests

The author(s) declare that they have no competing interests.

## Authors' contributions

HR conducted the basic study. OS contributed with the intellectual discussions and inputs in draft. ID and BC have discussed the results and statistical methods. AH, RS and NA read the script and have given their expert comments and suggestions to improve the study.

## Pre-publication history

The pre-publication history for this paper can be accessed here:



## Figures and Tables

**Table 1:** Socio-demographic characteristics of the sample

	**Pakistani (*n *= *339*)**	**Norwegian (*n *= *13581*)**
	**%**	**%**

**Age cohort**		
30 years	28.3	23.0
40/45 years	54.0	33.8
59/60 years	15.6	23.9
75/76 years	2.1	19.3
**Gender**		
Male	51.3	44.8
Female	48.7	55.2
Marital status*		
Married	90.6	47.3
Others	9.4	52.7
**Education**		
1–9 years	26.8	14.0
10–12 years	33.9	24.7
13+ years	39.2	61.3
**Employment**		
**status**		
With jobs	44.6	69.3
Without jobs	38.9	10.7
Missing	16.5	20.0

* Married category also includes registered partners

**Table 2:** Psychosocial variables by ethnicity. Means with 95% Confidence Interval adjusted for age and sex by ANOVA.

**Variables**	**Pakistani**		**Norwegian**		
	
	**number**	**Mean (95% CI)**	**number**	**Mean (95% CI)**	P* value
**Social support**					
Good friends	258	4.20 (3.85–4.58)	12323	6.95 (6.89–7.0)	<0.001
Other's interest	273	2.66 (2.56–2.76)	10612	3.03 (3.02–3.05)	<0.001
**Social participation**					
Participation in groups	243	0.88 (0.69–1.06)	12935	1.16 (1.13–1.18)	0.003
Influence on local community	263	1.76 (1.66–1.86)	10760	1.62(1.60–1.64)	0.007
**powerlessness**	120	2.53 (2.45–2.62)	11129	2.15 (2.14–2.16)	<0.001

* p value for ethnicity

**Table 3:** Association between psychological distress (dependent variable) and psychosocial factors, adjusted for age and sex, by ethnicity: findings from linear regression

**Variables**	**Psychological distress**					
	**Pakistanis**			**Norwegians**		
	**ß**	**95% CI**	**p**	**ß**	**95% CI**	**p**

**Social support**						
Good friends	-0.06	-0.09 to -0.03	<0.001	-0.03	-0.03 to -0.03	<0.001
Other's interest	-0.09	-0.16 to -0.01	0.021	-0.11	-0.12 to -0.10	<0.001
**Social participation**						
Participation in groups	0.06	-0.01 to 0.12	0.061	-0.03	-0.04 to -0.03	<0.000
Influence on local community	-0.02	-0.11 to 0.06	0.601	-0.04	-0.05 to -0.03	<0.001
**Powerlessness**	0.37	0.19 to 0.56	<0.001	0.25	0.23 to 0.26	<0.001

**Table 4:** The association between distress (dependent variable) and ethnic group (Norwegian as reference) in multiple regression analysis.

**Model**	**Psychological distress**			
	
	**ß**	**95% CI**	**p**	**Covariates**
Model 0	0.23	0.19 to 0.29	<0.001	Age and sex
Model 1	0.07	0.01 to 0.12	0.016	Age, sex, education and employment status
Model 2	0.12	0.07 to 0.18	<0.001	Age, sex, social support*
Model 3	0.00	-0.06 to 0.06	0.922	Age, sex, education, employment status, social support

* Number of friends and people's interest in your activities
